# Chagas screening and treatment among Bolivians living in Madrid, Spain: The need for an official protocol

**DOI:** 10.1371/journal.pone.0213577

**Published:** 2019-03-08

**Authors:** María Romay-Barja, Teresa Boquete, Obdulia Martinez, Marlene González, Débora Álvarez-Del Arco, Agustín Benito, Teresa Blasco-Hernández

**Affiliations:** 1 Centro Nacional de Medicina Tropical, Instituto de Salud Carlos III, Madrid, Spain; 2 Red de Investigación Colaborativa en Enfermedades Tropicales, RICET, Madrid, Spain; 3 Centro Nacional de Epidemiología, Instituto de Salud Carlos III, Madrid, Spain; Faculty of Science, Ain Shams University (ASU), EGYPT

## Abstract

**Background:**

It is estimated that around 52,000 people live with Chagas in Spain, but only 10% have been diagnosed. Migrants from Bolivia bear the burden of Chagas infection in Spain. However, little is known about their current management of Chagas diagnosis and treatment patterns. This study aimed to assess the Chagas related disease perception and health behaviour of Bolivians living in Madrid.

**Methods and principal findings:**

For a first time, a cross-sectional survey about Chagas’ knowledges and practices was carried out in Madrid, Spain. A total of 376 Bolivians were interviewed about their Chagas health-seeking behaviour. Differences between men and women were assessed Most of Bolivians living in Madrid have access to the public health services. 44% of Bolivians included in the survey had a Chagas screening test done. However, while women did their test for Chagas mostly at hospital (59.2%), men also used the community campaigns (17.5%) and blood banks (14.3%). The prevalence reported among Bolivians tested was 27.7%. Unfortunately, more than half of those reporting a positive test for Chagas did not begin or completed treatment. Only 45.7% of positives reported having had their children tested for Chagas.

**Conclusions:**

Despite the increase in the number of Chagas diagnoses done in Madrid, the number of Bolivians who tested positive and then started or completed treatment remains very low. The fact that most Bolivians’ access to the health system is through the primary healthcare services should be considered for improving management of cases and follow-up of treatment adherence. Local and national protocol establishing guidelines for the screening and treatment of Chagas disease would help improving case detection and management at all levels of the healthcare system.

## Introduction

Chagas disease is endemic in 21 continental Latin American countries, where almost 6 million people are infected with *Trypanosoma cruzi*,[1]. Considered one of the main neglected tropical diseases in Latin America, Chagas disease has crossed borders to North America and Europe, due to population mobility, where an estimated 120,000 people currently live with [2–4]. Endemically transmitted by *triatomine insects*, the main routes of transmission in non-endemic countries are congenital transmission, blood transfusion and solid organ transplants [5].

Presenting in two phases, Chagas disease manifests with an initial acute phase lasting two months, with high parasitemia, and is mostly asymptomatic. When untreated, the disease evolves into a chronic phase, with the parasites hidden in target tissues, especially the cardiac and digestive system muscles. Chagas disease remains the leading cause of cardiomyopathy and death from cardiovascular disease in patients’ ages 30 to 50 years, causing more than 10,000 deaths per year [2].

Available treatments (benznidazole and nifurtimox) have high efficacy during the acute phase and reduce the risk of disease progression in patients in the chronical stage of the disease (patients without evidence of cardiac or gastrointestinal disease) [6]. Efficacy is also especially high in congenitally infected newborns, with a cure rate of 100% [7]. However, efficacy of both medicaments diminishes the longer the infection has been present [8]. Early diagnosis is critical to improving outcomes for those living with Chagas and to prevent further vertical transmission.

An estimated 52,000 people in Spain live with Chagas, but only 10% have been diagnosed [9]. Migrants from Bolivia have the highest prevalence of the disease in Europe, at 18.1% [10]. They bear the burden of parasite infection in Spain, accounting for 81% of reported cases [5].

Since 2005, Spain has established legal requirements to ensure the safety of the blood supply and organ transplantation by monitoring tissues for Chagas disease [11]. However, only 3 of 17 autonomous regions (Valencia, Catalonia and Galicia) have an official protocol that recommends routine primary care testing in the population from endemic areas [12]. National strategies to increase screening uptake are nonexistent. Madrid does not have specific legislation addressing screening and treating Chagas. Different health professionals from different institutions have produced guidelines encouraging screening of pregnant women from endemic areas [13] but evidence suggests that adherence to these guidelines is poor [14].

Although the importance of social and cultural factors is broadly acknowledged, current approaches to neglected tropical diseases almost always overlook the socio-cultural aspects of Chagas [10]. The consequences are a limited understanding of the condition and obstacles to sustainable prevention and control [6]. Chagas disease occurs in specific contexts marked by sociocultural, political and economic circumstances [15]. The absence of symptoms, disease risk perceptions, the lack of information about available services, and immigration policies also play an important role in the health-seeking behaviour of the affected population in non-endemic countries [16,17].

Understanding this health-seeking behaviour is important to guide Chagas screening policies at the local and national levels. Associations with gender also need to be considered when talking about patterns of immigrant healthcare use. Usually, men are less likely to use health services while women have closer ties to the health system because of their reproductive role [18].

Little is known about the current Chagas related health-seeking behaviour of Bolivians in Spain. Previous behavioural studies have focused on positive cases [15,19] but not on the Bolivian general population. This study assessed Chagas-related health-seeking behaviour and disease perception among Bolivians living in Madrid to generate accurate information that may help strengthen interventions aimed at improving the screening and treatment of the affected population.

## Materials and methods

### Study area and population

This cross-sectional study was carried out March–August 2017 in Madrid, Spain. It was part of a project aimed at assessing access and use of health services among Bolivians with the diagnosis of Chagas disease in Madrid. The survey also aimed to provide information about the knowledge, attitudes and practices of the targeted population.

**Fig 1:**
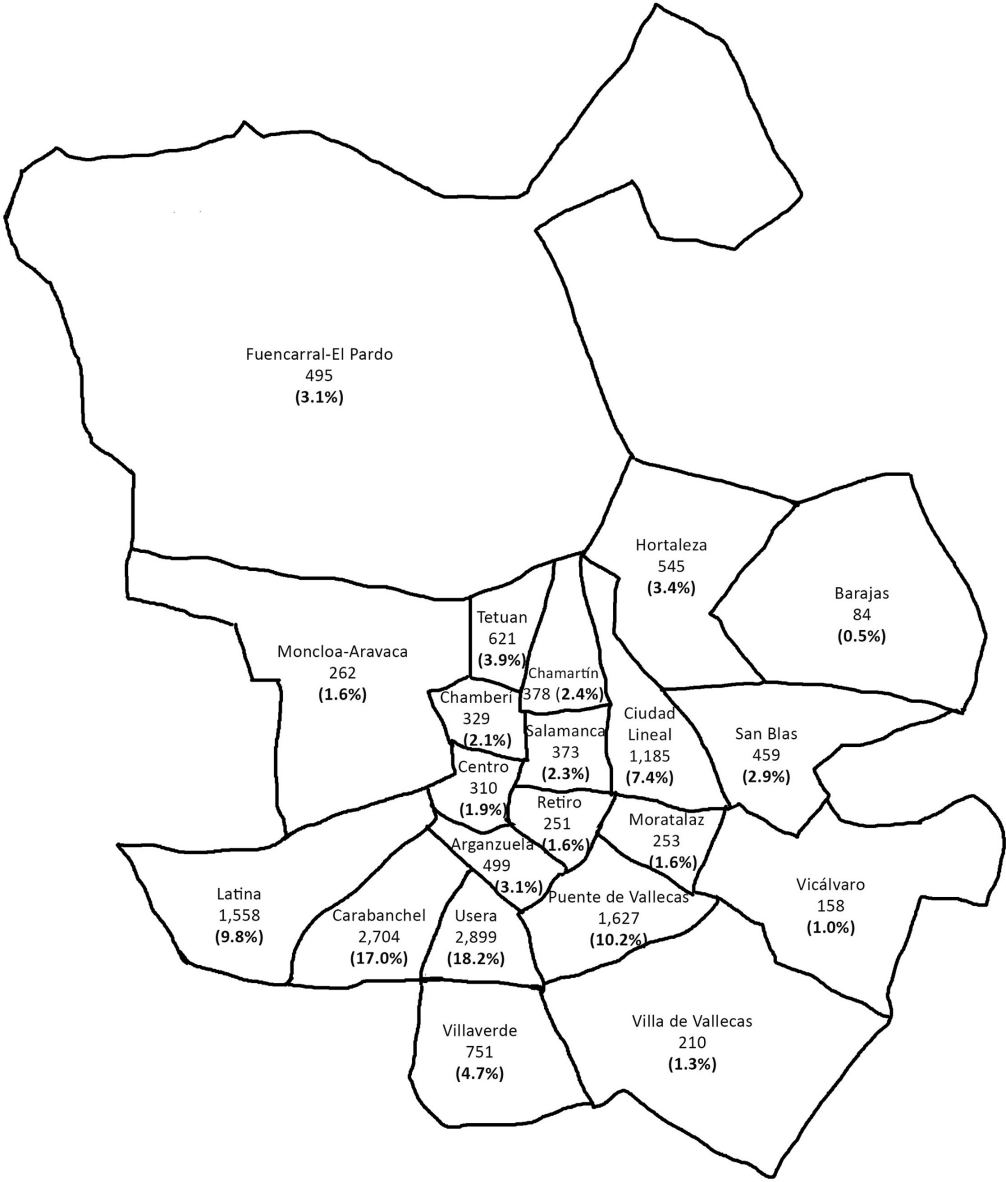
Geographical distribution of the Bolivian population in Madrid according to municipal census. Data from https://www.madrid.es/.

Based on the last municipal census [20], a total of 15,951 Bolivians (6,758 men and 9,193 women) live in Madrid, distributed principally in Usera, Carabanchel, Puente de Vallecas, and Latina neighborhoods (Fig 1).

The estimated prevalence of Chagas among Bolivians living in Madrid is around 25%, with a 35.5% overall rate of visceral involvement [14]. In April 2012, undocumented migrants (people without a Public Health Insurance (PHI) card) lost their universal healthcare access because a change in the law, and their access was reduced to the emergency services [21]. Since then, some public-private partnerships have been established to address these challenges and different non-profit organizations have implemented some education and screening community campaigns [14].

### Sampling and data collection

The study was a descriptive cross-sectional survey designed to determine whether Bolivians living in Madrid have adequate knowledge about Chagas screening and to compare by sex their attitudes and practices related to the screening and treatment of Chagas in Madrid.

A sample size of the Bolivian population living in Madrid was calculated considering 95% confidence level, 5% error rate, and Chagas disease knowledge rate of 50%. A total of 376 people were selected among Bolivians attending at the waiting room of the Bolivian Consulate in Madrid. A structured questionnaire was administered to participants according to the percentages of men and women in the population. Inclusion requirements were age over 18 years and have heard about Chagas.

Participants were asked about their health-seeking behaviour. They were interviewed about socioeconomic characteristics and factors related to their attitudes and practices regarding management of Chagas, care itineraries, screening and treatment. The questionnaire used was previously tested.

### Data analysis

A descriptive univariate analysis of participant characteristics was conducted using frequency tables for categorical variables. For normally or not-normally distributed continuous variables, we used mean and standard deviation or median and interquartile range, respectively. Differences in sociodemographic characteristics and treatment-seeking behaviours between men and women were assessed using the chi-squared test for independence for categorical variables. For normally or not-normally distributed continuous variables, we used the Student’s t test or the non-parametric Mann-Whitney test, respectively. *P values* < 0.05 were considered to be statistically significant. Data analyses were performed using STATA software version 15.

### Ethics statement

This study was approved by the Ethics Committee of the Spanish National Health Institute, Carlos III (CEI PI 50_2016). Interviewees gave written informed consent for participation in the study.

## Results

Of the 376 Bolivians interviewed about their Chagas-related health-seeking behaviour, 159 (42.4%) were men and 217 (57.6%) women. Table 1 summarizes the socioeconomic and demographic characteristics of the surveyed population. The participants had a mean age of 38 years (IQ: 33–45, minimum 18, maximum 77). Most of them (66.0%) were married or lived with a partner, but women were widowed or divorced more frequently than men (13.4% vs. 3.8%). Most of the Bolivians reported having completed secondary school or beyond (74.2%). Significantly more women (86.6%) than men (77.95) reported having children. The mean year of arrival in Spain was 2005 (IQ: 2003–2006, minimum 1982, maximum 2017) but a not-irrelevant number arrived later, especially among the men (49.7%).

**Table 1:** Socio-economic characteristics of Bolivians living in Madrid, by sex.

	Male		Female		*P-value*
	n = 159	%	n = 217	%	
**Age**					
18–24	15	9.43	14	6.45	
25–34	47	29.56	48	22.12	
35–44	56	35.22	87	40.09	
45–54	27	16.98	46	21.20	
55–64	11	6.92	12	5.53	
> 65	3	1.89	10	4.61	0.241
**Marital status**					
Partner	51	32.08	50	23.04	
Married	60	37.74	87	40.09	
Single	42	26.42	51	23.50	
Widow	3	1.89	8	3.69	
Divorced	3	1.89	21	9.68	0.011
**Education**					
Primary school or less	38	23.90	59	27.19	
Secondary school or more	121	76.10	158	72.81	0.471
**Children**					
No	35	22.01	29	13.36	
Yes	124	77.99	188	86.64	0.027
**Year of arrival**					
< 2000	4	2.52	4	1.84	
2000–2005	76	47.80	124	57.14	
2006–2010	56	35.22	71	32.72	
2011–2017	23	14.47	18	8.29	0.166
**Bolivian department**					
Cochabamba*	57	35.85	96	44.24	
Santa Cruz*	68	42.77	82	37.79	
La Paz	18	11.32	17	7.83	
Potosi	5	3.14	4	1.84	
Chuquisaca*	4	2.52	5	2.30	
Oruro	3	1.89	4	1.84	
Beni	1	0.63	6	2.76	
Tarija*	3	1.89	2	0.92	
Pando	0	0.00	1	0.46	0.469
**Area**					
Rural	42	26.42	58	26.73	
Urban	98	61.64	124	57.14	
Both	19	11.95	35	16.13	0.488
**House cosntruction in Bolivia**					
Adobe	31	19.50	58	26.73	
Brick	84	52.83	96	44.24	
Adobe and brick	18	11.32	38	17.51	
Brick and concrete block	16	10.06	14	6.45	
Concrete block	10	6.29	11	5.07	0.110
**Madrid District**					
Aganzuela	7	4.40	4	1.84	
Barajas	1	0.63	1	0.46	
Carabanchel	29	18.24	41	18.89	
Centro	2	1.26	9	4.15	
Chamartin	3	1.89	3	1.38	
Chamberí	3	1.89	4	1.84	
Ciudad Lineal	13	8.18	16	7.37	
Fuencarral	3	1.89	9	4.15	
Hortaleza	3	1.89	7	3.23	
Latina	10	6.29	21	9.68	
Moncloa	0	0.00	2	0.92	
Moratalaz	4	2.52	4	1.84	
Puente de Vallecas	19	11.95	22	10.14	
Retiro	3	1.89	1	0.46	
Salamanca	1	0.63	6	2.76	
San Blas	6	3.77	6	2.76	
Tetuan	11	6.92	6	2.76	
Usera	31	19.50	42	19.35	
Vicalvaro	0	0.00	1	0.46	
Villa de Vallecas	1	0.63	2	0.92	
Villaverde	9	5.66	10	4.61	0.515
**Are you currently working?**					
No	53	33.33	66	30.41	
Yes	106	66.67	151	69.59	0.548
Jobs**					
Managers	1	0.94	5	3.31	
Professionals	3	2.83	2	1.32	
Technicians	1	0.94	0	0.00	
Services and Sales	29	27.36	43	28.48	
Skilled agricultural, forestry and fishery	8	7.55	0	0.00	
Craft and related trades workers	29	27.36	0	0.00	
Plant and machine operators	8	7.55	0	0.00	
Elementary occupations	26	24.53	101	66.89	
Armed forces	1	0.94	0	0.00	0.000
**Household income**					
No one	5	3.14	7	3.23	
<1000 €	51	32.08	103	47.47	
1001–2000 e	81	50.94	86	39.63	
>2000 €	17	10.69	13	5.99	
Don't know	5	3.14	8	3.69	0.032

*Departments where Chagas is endemic in Bolivia

**According to ISCO classification

Regarding place of origin, 80.6% of the Bolivians came from the Cochabamba or Santa Cruz departments. Of the group, 59.0% were from an urban area and 47.9% lived in a house built with bricks, without differences between sexes.

Concerning their lives in Madrid, the interviewed Bolivians were distributed through the city districts similar to the municipal census. Most of them lived in the Usera (19.4%), and Carabanchel (18.65%) districts, followed by Puente de Vallecas (10.9%), Latina (8.2%) and Ciudad Lineal (7.7%). Regarding their economic situation, 68.4% of the Bolivians had a job at the time of the interview, but men and women differed significantly in occupations and household income. Women worked mainly in elementary occupations such as domestic cleaning and helpers (66.89%), whereas men were more often employed in services like refurbishment, building and related trade jobs (54.7%). Of the women, 50.7% reported living with less than 1000€ as their household monthly income, while 61.6% of men reported earning more.

### Health-seeking behaviour

Most of the Bolivians interviewed hold a PHI card (87.0%), and 75.8% reported to have not had any problem to go to the doctor in Spain (Table 2). When they felt unwell, the most often used health service was the Primary Health Centre, but men and women did differ (91.7% of women vs 83.6% of men, *P* = 0.013). Men tended to self-medicate or go to the pharmacy more frequently than women. Most of Bolivians (80.6%) were aware that screening for Chagas is available in Spain at health centres (35.6%) or hospitals (54.8%).

**Table 2:** General health seeking behaviour and perceptions.

	Male		Female		*P-value*
	n = 159	%	n = 217	%	
**If you feel ill, where you go?**					
Health Center	133	83.65	199	91.71	0.016
Hospital emergency room	20	12.58	25	11.52	0.755
Self-medicate	17	10.69	16	7.37	0.261
Pharmacy	2	1.26	0	0.00	0.098
**Problems to go to the doctor in Spain**					
No	119	74.84	166	76.50	
Yes	40	25.16	51	23.50	0.711
**Public Health Insurance card**					
No	24	15.09	25	11.52	
Yes	135	84.91	192	88.48	0.309
**It is possible to do be tested for Chagas in Spain**					
No	2	1.26	3	1.38	
Yes	125	78.62	178	82.03	
Don't know	30	18.87	31	14.29	0.600

### Chagas diagnosis

**Fig 2:**
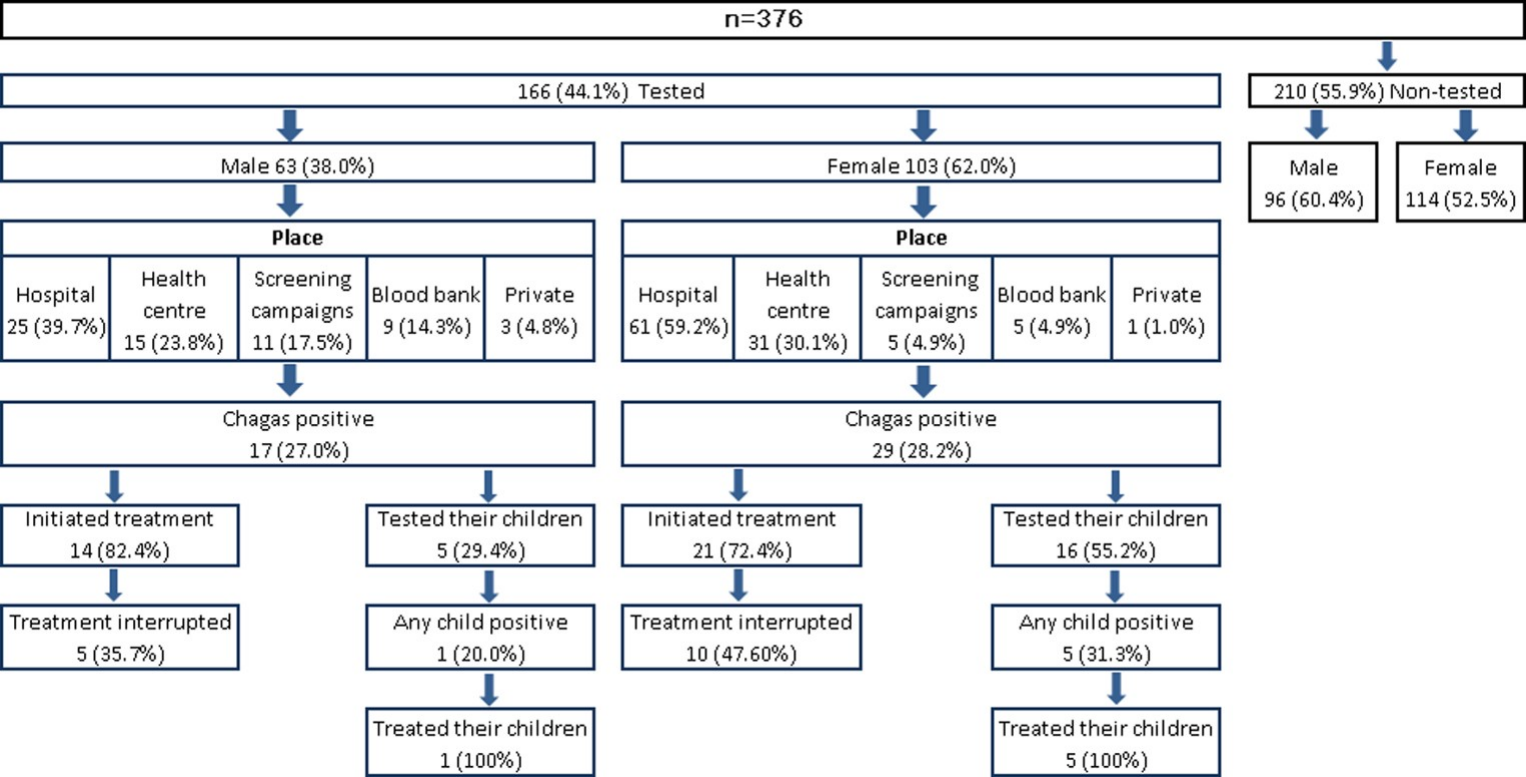
Chagas diagnosis and treatment respondents’ behaviour.

Fig 2 shows the Chagas diagnosis and treatment behaviour of Bolivians living in Madrid. Only 44.1% (166) of Bolivians surveyed had done their Chagas screening. However, men and women differed in where they had their Chagas test performed (*P* = 0.003). Overall, the most mentioned places where the hospital and primary health centre. But women chose a hospital more often for testing (59.2%), while men also chose the community screening campaigns (17.5%) and blood banks (14.3%).

Among interviewees who had done their diagnostic testing, almost all reported having had no difficulty, and 70% reported having been advised to do so (Table 3). The source of the recommendation differed between the sexes, with 56.8% of women following the recommendation of a doctor but only 30.2% of men doing so (*P* = 0.005). Men also followed the advice of a familiar or at community campaigns more frequently than women (*P =* 0.052).

**Table 3:** Perceptions and practices of those who have had Chagas screening.

	Male		Female		*P-value*
	n = 63	%	n = 103	%	
**Difficulties in getting tested for Chagas**					
No	63	100.0	100	97.1	0.212
At work	0	0.0	1	1.0	0.577
My doctor did not know what to do	0	0.0	2	1.9	0.322
**Received any advice to do the test**					
No	20	31.7	29	28.2	
Yes	43	68.3	74	71.8	0.531
**Sources of advice**					
Doctor	13	30.2	42	56.8	0.005
Family	11	25.6	10	13.5	0.101
Friends	3	7.0	8	10.8	0.493
Campaigns	6	14.0	3	4.1	0.052
Blood banks	3	7.0	4	5.4	0.730
Other	7	16.3	7	9.5	0.273

### Chagas prevalence and treatment behaviour

The prevalence Chagas among the Bolivians who have been screening was 27.7%, with not significant differences between the sexes. Among those positive for Chagas, 63.0% were women, between 35–44 years old (50%) with no positive cases under 31 years old. Most of them came from a rural area (41.3%) of Santa Cruz or Cochabamba (47.8% and 41.3% respectively), where they lived in a house of adobe (50.0%) or mixed, adobe and brick (21.7%), and have seen a Vinchuca (89%).

Almost 24.0% of the Bolivians positives for Chagas reported not having initiated treatment (Fig 2). Men and women further differed about their reasons. Among the women, the principal reasons cited for not having done so were “I do not have time” (37.5%) and “I feel fine” (25.0%). Men cited “I feel fine” most often (66.7%).

Among those who did start treatment, 42.9% interrupted it mainly because of side-effects (90.0% of women vs 40.0% of men) or because they moved (10% of women vs 40.0% of men). Regarding which treatment they received, 25 (71.4%) did not remember, 9 people said benznidazole, and only one woman mentioned nifurtimox.

Only 45.7% of the group testing positive for Chagas had had their children tested. Women had done so more frequently than men, but the difference was not significant. However, 100% of Bolivians whose children had tested positive for Chagas reported having provided their children treatment.

### Perceptions of non-tested Bolivians

Table 4 shows the perceptions and practices of the non-tested Bolivians. Asked if they had received any advice about having Chagas screening since arriving in Spain, only 30.7% of women and 13.5% of men reported having received such advice. Among those who have been advised, only two (4.1%) received that advice from a medical doctor.

**Table 4:** Perceptions and practices of untested Bolivians.

	Male		Female		*P-value*
	n = 96	%	n = 114	%	
**Received any advice to do the test**					
No	83	86.46	79	69.30	
Yes	13	13.54	35	30.70	0.003
**Sources of advice**					
A familiar	5	5.21	21	18.42	0.014
A friend	4	4.17	12	10.53	0.817
NGO	2	2.08	1	0.88	0.111
Someone with Chagas	1	1.04	3	2.63	0.920
My doctor	1	1.04	1	0.88	0.456
**You will do the test soon?**					
No	49	51.04	81	71.05	
Yes	39	40.63	33	28.95	
I'll think about it	8	8.33	0	0.00	0.001
**Why not**					
As long as I do not have symptoms	22	22.92	11	9.65	0.007
Until the doctor will decide	0	0.00	6	5.26	0.024
I'm not a risk of having it	12	12.50	10	8.77	0.516
Lack of time	2	2.08	2	1.75	0.622
It is better not to know	3	3.13	4	3.51	0.594

Differences between sexes were detected regarding reasons for not being tested (*P* = 0.010). Men more frequently mentioned not having symptoms (34.4%) than did women (21.0%). Thinking that they did not have Chagas was the second most mentioned reason for both sexes (22.9% men and 20.2% women). Asked if they thought that they would have their Chagas test soon, 61.9% of non-tested Bolivians said no, more of them women.

## Discussion

This study offers new aspects of Bolivians’ health-seeking behaviour in Madrid, their Chagas screening, treatment practices and related attitudes. Despite the homogeneity of the sample, men and women had important differences in their behaviours that should be considered in the design of strategies to improve access of the endemic population to the diagnosis and treatment of Chagas.

According to studies on migratory flows, Bolivian migration to Spain has been especially intense since 2002, although declining from April 2007 due to the visa requirement for entering the EU [22]. This pattern is confirmed in our study, with most participants having arrived between 2003 and 2006. Differences between men and women in the year of arrival are explained by the fact that this migration presents an important feminization, especially in its beginnings [23]. The age, regions of origin and labour occupations by sex of our surveyed population match with data recorded in the National Survey of Immigrants [24]. Most of the Bolivians interviewed hold a PHI card which implies that most of them have a stable status. This situation would represent an improvement since 2007 [24] and may be related to the amount of time living in Spain, which usually was 12 years or more. However, a 24% of interviewed reported having problems going to the doctor, mainly because they did not hold a PHI card at that time or were under work constraints [25,26].

Other aspects that imply an improvement in their integration is that most Bolivians interviewed reported attending the primary level of health services when they felt unwell, with significant differences by sex. As for other migrants, men tended to self-medicate more frequently, while women were more likely to use the health system services [15]. Another improvement was that most of Bolivians knew that it is possible to have Chagas screening in Madrid through the public health service. The lack of knowledge about the public health system was one of the main barriers to diagnosis they encountered when they first arrived[27].

However, less than half of Bolivians reported to having been screened. Although a 44% test rate should be considered insufficient, it represents a marked improvement over previous estimations of only 10% [9]. This study did not found significant differences in the reported screening rate by sex, even though mothers and their newborns have been the most targeted population for Chagas interventions in Madrid [27],. The existence of a professional consensus document about screening women [13] does not seem to have been sufficient for reaching them. Furthermore, only half of women who tested positive reported having had their children tested. This low rate of child screening confirms a limited awareness of the vertical transmission of Chagas in the Bolivian population and of the benefits of early screening [19]. Further educational campaigns efforts should target improving Bolivians’ knowledge about vertical transmission and the benefits of early child screening. In Spain, it appears that it would be cost-efficient to implement a screening program to control Chagas disease in the general Bolivian population because of the higher prevalence, and not only in mothers and their relatives in case of a positive result[28]. Regardless of the strategy in Madrid, there is a clear need for official protocols to reach this population.

Despite using primary health services when they feel unwell, most women had their testing done in the hospital or health centre, while men also had it done at the community screening campaigns and at blood banks. In order to avoid barriers, if Bolivians in Madrid access the health service through primary care, this entry point should be their pathway to Chagas screening. Unfortunately, being screened in Madrid is more of an individual decision than a public health policy [13].

The low institutional involvement in Chagas screening is also reflected in who recommended it. Less than half of Bolivians screened reported having done so because of a medical recommendation. While doctors seemed to guide more women to screening as results of pregnancy, men mainly were advised by their family to do so. Much more work is needed at the institutional level to improve Chagas diagnoses advice and screening coverage.

The prevalence observed among those who reported have been tested is similar to values reported in other studies [14] as well as the characteristics of those positive for Chagas [29]. The main problem in Madrid is that more than a half of those who test positive have not begun or completed therapy. Chagas treatment adherence seems to have remained low in recent years in Madrid. Rates of treatment discontinuation are higher than those found in previous studies [30], while adverse reactions to medication continue to be the main cause of treatment interruption[30,10]. Improving follow-up on positive Chagas patients and their treatment adherence must be a cornerstone of the Chagas control in Madrid. Strategies similar to those used in other diseases like Directly Observed Therapy (DOT), phone-based encounters and the involvement of patient associations have been linked with Chagas treatment adherence improvement elsewhere [31].

Bolivians who were untested largely said that they had not received any advice to be tested. Those who have received such advice got it mainly from family or friend and not from a doctor. Again, the lack of an institutional strategy to inform at risk population about the advantages of Chagas screening seems to affect the final decision about being tested. Among those who were untested, women were significantly more reluctant than men to say that they planned to have the testing soon. This result is especially relevant for its implications for the congenital Chagas. As in endemic countries, the main reason for not getting tested was the absence of symptoms [32,33]. Other studies have also found that fear of treatment side-effects and an uncertain outcome are reasons for not doing the test [17]. To reach the reluctant population, the educational campaigns should focus on promoting a change in perception among the Bolivian population, engendering a more positive attitude about diagnosis and treatment benefits, especially among women of reproductive age and their children.

This study has some limitations. First, it was a cross-sectional study conducted in Madrid, so the findings might not generalize to very different contexts. Second, recall accuracy about their screening and their results could be a problem, especially if the test was negative.

## Conclusions

Despite the increase in the number of Chagas diagnoses done in Madrid, the number of Bolivians who tested positive and then started or completed treatment remains very low. Also, the number of children tested from positive mothers is low. Being diagnosed with and treated for Chagas is still a personal decision instead of a public health policy. An official protocol is needed to establish guidelines for the control and treatment of Chagas disease in Madrid. The fact that most Bolivians’ access to the health system is through the primary healthcare services should be considered when it comes to improving follow-up of treatment adherence.

## Supporting information

S1 Questionnaire Questionnaire in Spanish. (PDF)

S2 Questionnaire Questionnaire in English. (PDF)
