# Supplementary material for: Chagas screening and treatment among Bolivians living in Madrid, Spain: The need for an official protocol
Source: PLoS One. 2019 Mar 8;14(3):e0213577. doi: 10.1371/journal.pone.0213577 (PMC6407767; doi:10.1371/journal.pone.0213577)
Supplement: S1 Questionnaire — (PDF) [file pone.0213577.s001.pdf]

B. PERCEPCIONES Y PRÁCTICAS RESPECTO A LA ENFERMEDAD DE CHAGAS

Vamos a hablar sobre sus percepciones y prácticas. Por favor, es muy importante que conteste con sinceridad. No hay respuestas equivocadas, todas sus respuestas son adecuadas.

B.1 Cuando se siente enfermo, ¿A dónde va usted?

Acudir al centro de salud.......................................1
Acudir al hospital a urgencias 2
Médico privado 3
A la farmacia..4
Otros (especificar) 98
N.S…………………………………………………………………101

B.2 ¿Ha tenido usted alguna vez problemas para ir al médico en España?

Sí…………………………………………………1
No…………………………………………………2
N.S…………………………………………101

B.3. ¿Ha visto usted alguna vez una vinchuca?

Sí……………………………………….....1 → **Especificar dónde**___________________________________
No……………………………………..…2
N.S…………………………………101

B.4. ¿Y usted sabe si se puede hacer la prueba del Chagas en España?

Sí se puede…………………………………1 → **Especificar dónde:**
____
No se puede…………………………….…2
N.S……………………………………….…101

B.5. ¿Se ha hecho usted la prueba del Chagas?

Sí 1
No……………………………………………………………………2 → **Pasa a B.16**
N.S……………………………………………………………….101→**Pasa a B.16**
N.C………………………………………………………………….99→**Pasa a B.16**

B. 6. ¿Y en qué país fue?

España 1
Bolivia .... 2→ **Pasa a B.11**
España y Bolivia………………………………………………….3
Otros (especificar) ....98→ **Pasa a B.11**
N.S .... 101→ **Pasa a B.11**

B.7 En España, ¿Dónde acudió a hacerse la prueba?

Al hospital...1
Centro de Salud...2
Médico privado 3
Asociación 4
Otros (especificar) 98
N.S 101

B.8 ¿Le aconsejó alguien para hacerse la prueba de Chagas?

Sí...1
No...2 → **Pasa a B.10**
N.S..101 → **Pasa a B.10**

B.9. ¿Quién?

Mi médico...1
Un familiar..4
Un amigo 5
En la asociación 6
Otros (especificar) 98
N.S 101

B.10. ¿Encontró alguna dificultad para hacerse la prueba?

Ninguna 1
No sabía dónde ir 2
No puedo faltar al trabajo.....................................3
No tenía tarjeta 4
Otros (especificar) 98
N.S 101

B.11. ¿Cuál fue el resultado de la prueba?

Positivo (tengo Chagas)..1
Negativo (no tengo Chagas) ....2 **(Fin de la encuesta)**
N.S ....101 **(Fin de la encuesta)**
N.C ....99 **(Fin de la encuesta)**

B.12 ¿Usted ha seguido o está siguiendo ahora el tratamiento para el Chagas?

Sí, en el pasado...1 → **Pasa a B.12.1**
Sí, lo estoy tomando actualmente...................... 2 → **Pasa a B.12.1**
No...3 → **Pasa a B.12.2**
Otros (especificar)... 98 → **Pasa a B.12.1**

B.12.1 ¿Qué tratamiento? (especificar):____
____

B.12.2. ¿Por qué? (especificar): ____

____

B.13. ¿Y ha interrumpido en algún momento el tratamiento?

Sí……………………………………………………………………1 → **Pasa a B.13.1**
No…………………………………………………………………2 **(Fin de la encuesta)**
N.S…………………………………………………………….99 **(Fin de la encuesta)**

B.13.1. ¿Por qué? : ____
____

B.14. Si tiene hijos, ¿Le ha hecho la prueba a sus hijos?

Sí……………………………………………………………………1 → **Pasa a B.15**
No ………………………………………………………2 **(Fin de la encuesta)**
N.S……………………………………………………………….99**(Fin de la encuesta)**

B.15. ¿Han tomado tratamiento sus hijos?

Sí……………………………………………………………………1 **(Fin de la encuesta)**
No…………………………………………………………… …2 **(Fin de la encuesta)**
No tienen Chagas…………………………………… 3 **(Fin de la encuesta)**
N.S……………………………………………………………….99 **(Fin de la encuesta)**

B.16. ¿Y por qué no se ha hecho la prueba?

No puedo faltar al trabajo......................................1
Nunca tengo síntomas 2
No tengo tarjeta sanitaria 3
Otros (especificar) 98
N.C……………………………………………………………………..99

B.17 ¿Alguien le ha aconsejado que se haga la prueba?

Sí…………………………………………………………………………1
No……………………………………………………………………….2 → **Pasa a B.19**
N.S………………………………………………………………101 → **Pasa a B.19**

B.18 ¿Quién o quienes se lo aconsejaron?

Mi médico 1
Un familiar...2
Un amigo 3
En una asociación...4
Otros (especificar) 98
N.S 101

B.19 ¿Cree que se hará la prueba próximamente?

Sí 1
No…………………………………………………………………………2
N.S. .... …….101

B.20 ¿Por qué? ____
____
