# Supplementary material for: Chagas screening and treatment among Bolivians living in Madrid, Spain: The need for an official protocol
Source: PLoS One. 2019 Mar 8;14(3):e0213577. doi: 10.1371/journal.pone.0213577 (PMC6407767; doi:10.1371/journal.pone.0213577)
Supplement: S2 Questionnaire — (PDF) [file pone.0213577.s002.pdf]

B. PRACTICES AND PERCEPTIONS ABOUT CHAGAS

Now we are going to talk about what you know and belief. Please, it is very important that you answer sincerely. There are no wrong answers; all your answers are good.

B.1 When you feel ill, Where you go?

Health Center…………………………………………………….1
Hospital emergency room......................................2
Private doctor..3
Pharmacy..4
Other (speciy) 98
D.N…………………………………………………………………….101

B.2 Have you ever had problems to go to the doctor in Spain?

Yes…………………………………………………1
No…………………………………………………2
D.N…………………………………………101

B.3. Have you ever see a vinchuca?

Yes…………………………………....1 → Specify **where**___________________________________
No…………………………………….2
N.S……………………………….101

B.4. Do you know if it is possible to be tested for Chagas in Spain?

Yes, it is possible……………………………….1 → **Specify where**
____
No, it is not possible……………………………..2
D.N………………………………………….101

B.5. Have you been tested from Chagas?

Yes 1
No………………………………………………………………………2 → go to **B.16**
D.N………………………………………………………………….101→Go to **B.16**
N.A. ………………………………………………………………….99→Go to **B.16**

B. 6. Which country?

Spain 1
Bolivia………………………………………………………………..2→ Go to **B.11**
Both………………………………………………………………………3
Other (specify) ....98→ Go to **B.11**
D.N...101→ Go to **B.11**

B.7 In Spain, Where did you go to be tested?

Hospital 1
Health Center...2
Private doctor..3
NGO 4
Other (specify) 98
D.N.. 101

B.8 Did you received any advice to do the test?

Yes...1
No..2 → Go to **B.10**
N.S..101 → Go to **B.10**

B.9. From who?

Doctor..1
A familiar 4
A friend..5
NGO 6
Other (specify) 98
D.N.. 101

B.10. Did you have any difficulty in getting tested for Chagas?

No one 1
I did not know where to go 2
I cannot miss work 3
I did not hold a PHI card 4
Other (speciy) 98
D.N.. 101

B.11. What was the result of your test?

Positive (I have Chagas)..1
Negative (I do not have Chagas) .... 2 **(END)**
D.N.. 101**(END)**
D.A .... 99 **(END)**

B.12 Have you followed treatment?

Yes, I did...1 → go to **B.12.1**
Yes, I'm currently in treatment............................... 2 → go to **B.12.1**
No..3→ go to **B.12.2**
Other (specify).. 98 → go to **B.12.1**

B.12.1 Which treatment? (specify):____
____

B.12.2. Why? (specify): ____

____

B.13. Have you ever stopped your treatment?

Yes………………………………………………………………1 → Go to **B.13.1**
No…………………………………………………………………2 **(END)**
N.S…………………………………………………………….99 **(END)**

B.13.1. Why?: ____
____

B.14. If he/she had children, Have you done the test to your children?

Yes………………………………………………………………1 → Go to **B.15**
No ………………………………………………………2 **(END)**
N.S…………………………………………………………………99 **(END)**

B.15. Have your children follow treatment?

Yes………………………………………………………………1 **(END)**
No……………………………………………………………… …2 **(END)**
They were negative ……………………………… 3 **(END)**
D.N……………………………………………………… …99 **(END)**

B.16. Why have you not been tested from Chagas?

I cannot miss work 1
Nunca tengo síntomas 2
Ido not have PHI card 3
Other (specify) 98
N.A…………………………………………………………………..99

B.17 Did you receive any advice to do test?

Yes…………………………………………………………………….1
No……………………………………………………………………..2 → Go to **B.19**
D.N……………………………………………………………….101 →Go to **B.19**

B.18 From whom?

My doctor 1
A familiar 2
A friend...3
NGO 4
Other (specify) 98
N.S 101

B.19 Do you think that you will do the test soon?

Yes 1
No…………………………………………………………………………2
N.S. .... …….101

B.20 Why? ____
____
